# Supplementary material for: Rational Modulation of Plant Root Development Using Engineered Cytokinin Regulators
Source: ACS Synth Biol. 2025 Jul 30;14(8):3013–23. doi: 10.1021/acssynbio.5c00051 (PMC12362619; doi:10.1021/acssynbio.5c00051)
Supplement: Supplementary file 1 [file sb5c00051_si_001.pdf]

Supporting Information for

Rational modulation of plant root development using engineered cytokinin regulators

Authors: Rohan Rattan[1,2,4,✤], Simon Alamos[1,2,3,✤], Matthew Szarzanowicz[1,2,3], Kasey Markel[1,2,3], Patrick M. Shih[1,2,3,5,*]

Affiliations:
[1]Joint BioEnergy Institute, 5885 Hollis Street, Emeryville, CA 94608, USA.
[2]Environmental Genomics and Systems Biology Division, Lawrence Berkeley National Laboratory, Berkeley, California, USA
[3]Department of Plant and Microbial Biology, University of California, Berkeley, CA 94720, USA
[4]Department of Bioengineering, University of California, Berkeley, California, USA
[5]Innovative Genomics Institute, Berkeley, California, USA
✤ These authors contributed equally
* Corresponding author. Email: pmshih@berkeley.edu

This PDF file includes:

Figure S1, S2

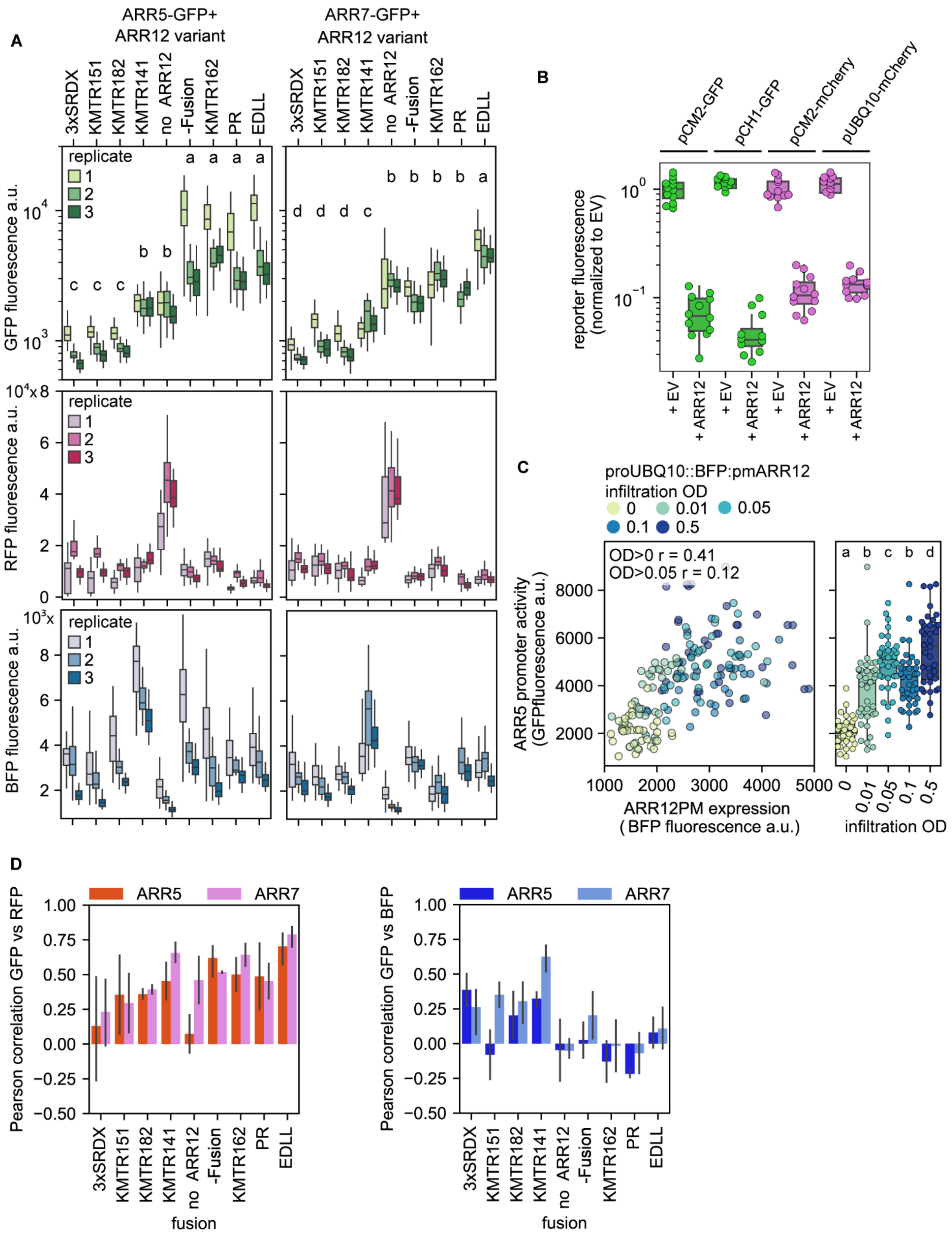
A
ARR5-GFP+
ARR12 variant
ARR7-GFP+
ARR12 variant
3xSRDX
KMTR151
KMTR182
KMTR141
no ARR12
-Fusion
KMTR162
PR
EDLL
replicate
1
2
3
GFP fluorescence a.u.
RFP fluorescence a.u.
BFP fluorescence a.u.
a
b
c
d
B
pCM2-GFP
pCH1-GFP
pCM2-mCherry
pUBQ10-mCherry
reporter fluorescence
(normalized to EV)
+ EV
+ ARR12
C
proUBQ10::BFP:pmARR12
infiltration OD
0
0.01
0.05
0.1
0.5
OD>0 r = 0.41
OD>0.05 r = 0.12
ARR5 promoter activity
(GFPfluorescence a.u.)
ARR12PM expression
(BFP fluorescence a.u.)
infiltration OD
D
ARR5
ARR7
Pearson correlation GFP vs RFP
Pearson correlation GFP vs BFP
fusion

Fig. S1: Characterization of pmARR12 variants for a range of transcriptional strength. (A) Top: Box plots showing the measured GFP output in *N. benthamiana* leaf discs for each BFP-pmARR12 fusion variant in 3 different replicates (n = 36 leaf disks per variant per replicate). Middle: signal in the RFP channel driven by the internal normalization strain. Bottom: signal in the BFP channel corresponding to the expression level of BFP-pmARR12 fusion variants. **(B)** Expression of ARR12 affects the normalization signal. Box plots showing the fluorescence of leaves infiltrated with GFP and RFP reporters driven by different constitutive promoters in combination with an empty vector (pCambia1300, labeled no ARR12) or the -Fusion BFP-pmARR12 variant. For each promoter, fluorescence was normalized to the mean EV fluorescence intensity. **(C)** Titration of the -Fusion BFP-pmARR12 variant shows limited concentration-dependence. The ARR12 effector strain was infiltrated at varying ODs in combination with a constant OD of the proARR5 reporter strain. Left: scatter plot of GFP vs BFP fluorescence. Right: box plots of GFP fluorescence for each ARR12 OD. **(D)** RFP, but not BFP, correlates well with reporter activity. Shown is the mean ± SD Pearson correlation between the signals of RFP and GFP (left) or BFP and GFP (right) across leaf punches for each variant and reporter combination. Box plots in (A)-(C) show the median ± IQR, whiskers show the minima and maxima excluding outliers, if present. For the GFP signal in (A), a Kruskal-Wallis test was run on each promoter dataset to determine if there were significant differences between variants, resulting in P = 1.1446e-119 for ARR5 and P = 4.1337e-112 for ARR7. The letters on top of each variant represent statistically significant groups (P < 0.05) based on Dunn's test.

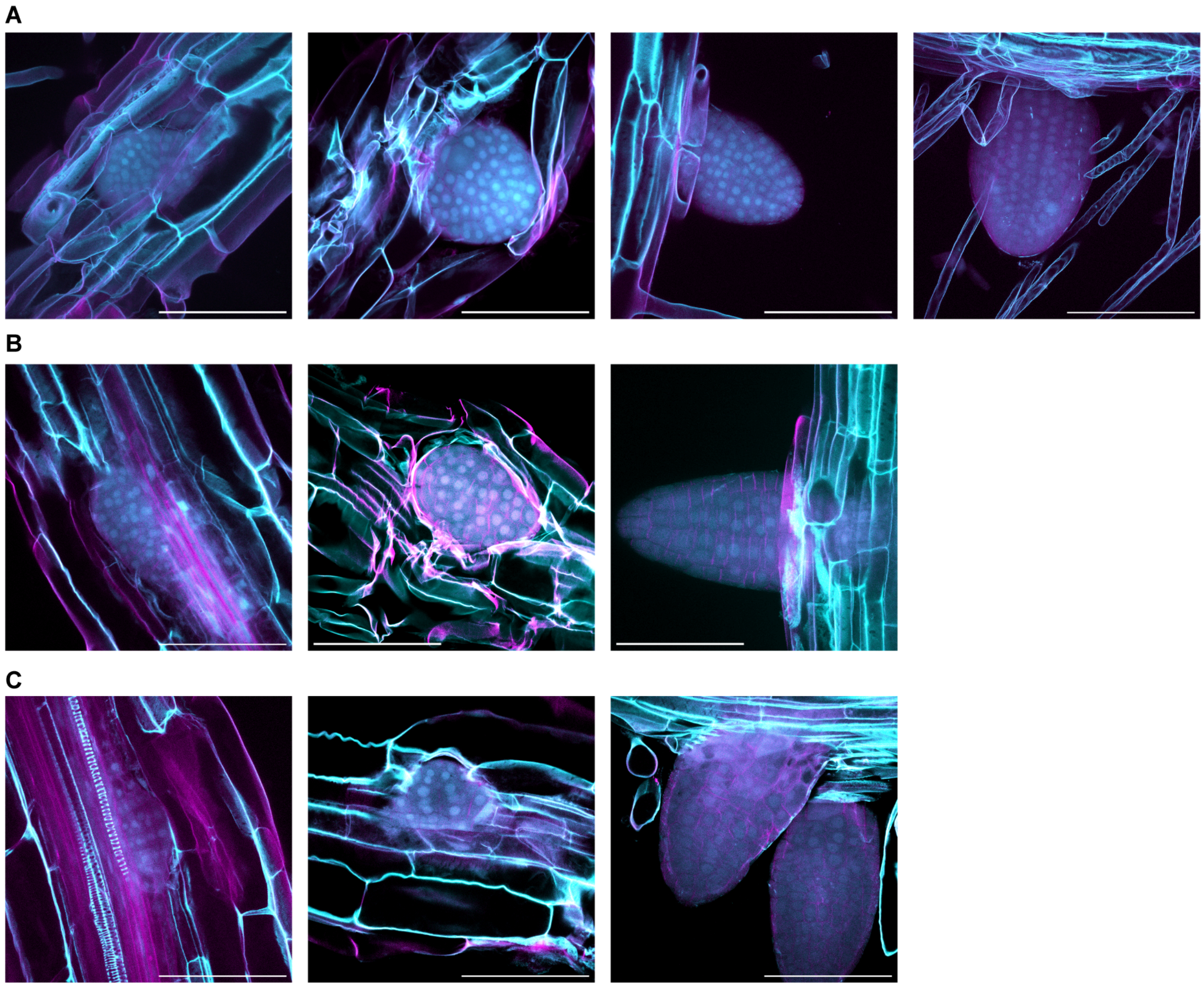

Figure S2: Localization of BFP-pmARR12-3xSRDX driven by the proGATA23 promoter in Arabidopsis seedlings. (A)-(C) Maximum projection confocal microscopy fluorescence images of proGATA23::BFP:pmARR12:3xSRDX plants. Cyan corresponds to BFP and cell wall autofluorescence. Magenta corresponds to the cell wall dye Direct Red 23. Figures (A), (B), and (C) each show details from T2 seedlings derived from T1 lines 1, 3, and 5, respectively. Scale bar = 100 µm.
